# Transcriptomics reveals transient and dynamic muscle fibrosis and atrophy differences following spinal cord injury in rats

**DOI:** 10.1002/jcsm.13476

**Published:** 2024-05-19

**Authors:** Hui Jean Kok, Drew B. Fletcher, Jacob C. Oster, Christine F. Conover, Elisabeth R. Barton, Joshua F. Yarrow

**Affiliations:** ^1^ Department of Applied Physiology and Kinesiology College of Health and Human Performance, University of Florida Gainesville FL USA; ^2^ Research Service, Malcolm Randall Department of Veterans Affairs Medical Center North Florida/South Georgia Veterans Health System Gainesville FL USA; ^3^ Division of Endocrinology, Diabetes and Metabolism College of Medicine, University of Florida Gainesville FL USA; ^4^ Brain Rehabilitation Research Center, Malcolm Randall Department of Veterans Affairs Medical Center North Florida/South Georgia Veterans Health System Gainesville FL USA; ^5^ Eastern Colorado Geriatrics Research, Education, and Clinical Center Rocky Mountain Regional Veterans Affairs Medical Center, VA Eastern Colorado Health Care System Aurora CO USA

**Keywords:** disuse atrophy, extracellular matrix remodelling, muscle fibre‐type shift, RNA‐sequencing, soleus muscle wasting, spinal cord injury

## Abstract

**Background:**

The rate and magnitude of skeletal muscle wasting after severe spinal cord injury (SCI) exceeds most other disuse conditions. Assessing the time course of molecular changes can provide insight into the progression of muscle wasting post‐SCI. The goals of this study were (1) to identify potential targets that may prevent the pathologic features of SCI in soleus muscles and (2) to establish therapeutic windows for treating these pathologic changes.

**Methods:**

Four‐month‐old Sprague–Dawley male rats received T9 laminectomy (SHAM surgery) or severe contusion SCI. Hindlimb locomotor function was assessed weekly, with soleus muscles obtained 1 week, 2 weeks, 1 month and 3 months post‐surgery (*n* = 6–7 per group per timepoint). RNA was extracted from muscles for bulk RNA‐sequencing analysis (*n* = 3–5 per group per timepoint). Differentially expressed genes (DEGs) were evaluated between age‐matched SHAM and SCI animals. Myofiber size, muscle fibre type and fibrosis were assessed on contralateral muscles.

**Results:**

SCI produced immediate and persistent hindlimb paralysis, with Basso–Beattie–Bresnahan locomotor scores remaining below 7 throughout the study, contributing to a progressive 25–50% lower soleus mass and myofiber atrophy versus SHAM (*P* < 0.05 at all timepoints). Transcriptional comparisons of SCI versus SHAM resulted in 184 DEGs (1 week), 436 DEGs (2 weeks), 133 DEGs (1 month) and 1200 DEGs (3 months). Upregulated atrophy‐related genes included those associated with cell senescence, nuclear factor kappa B, ubiquitin proteasome and unfolded protein response pathways, along with upregulated genes that negatively influence muscle growth through the transforming growth factor beta pathway and inhibition of insulin‐like growth factor‐I/Akt/mechanistic target of rapamycin and p38/mitogen‐activated protein kinase signalling. Genes associated with extracellular matrix (ECM), including collagens, collagen crosslinkers, proteoglycans and those regulating ECM integrity, were enriched within upregulated DEGs at 1 week but subsequently downregulated at 2 weeks and 3 months and were accompanied by >50% higher ECM areas and hydroxyproline levels in SCI muscles (*P* < 0.05). Myofiber remodelling genes were enriched in upregulated DEGs at 2 weeks and 1 month and were downregulated at 3 months. Genes that regulate neuromuscular junction remodelling were evident in muscles post‐SCI, along with slow‐to‐fast fibre‐type shifts: 1 week and 2 weeks SCI muscles were composed of 90% myosin heavy chain (MHC) type I fibres, which decreased to only 16% at 3 months and were accompanied by 50% fibres containing MHC IIX (*P* < 0.05). Metabolism genes were enriched in upregulated DEGs at 1 month and were further enriched at 3 months.

**Conclusions:**

Our results substantiate many known pathologic features of SCI‐induced wasting in rat skeletal muscle and identify a progressive and dynamic transcriptional landscape within the post‐SCI soleus. Future studies are warranted to consider these therapeutic treatment windows when countering SCI muscle pathology.

## Introduction

Limb paralysis from severe spinal cord injury (SCI) induces a loss of skeletal muscle mass and strength that exceeds most other disuse and denervation conditions in both magnitude and onset. To date, there are no clinically approved pharmacologic approaches to counter skeletal muscle atrophy associated with severe SCI, as several strategies to prevent the detrimental effects of SCI‐induced muscle wasting have been unsuccessful.^1^ A major challenge for effective anabolic therapies is how rapidly atrophy occurs, affording only a narrow window of opportunity to prevent loss of muscle mass. Further, atrophy associated with SCI requires multi‐pronged approaches that not only enhance anabolic actions but also prevent catabolic drive. Last, the complexity of the molecular pathways that underlie SCI‐induced atrophy is still being elucidated. Thus, there is a clear need to identify pathways that govern muscle wasting and remodelling after SCI, as well as when these pathways are most active at the transcriptional or activity levels, in order to optimize therapies for individuals living with SCI.

Molecular signatures of atrophy in individuals with SCI are available with a focus on a single timepoint, either a few days after injury or in chronic cases.^2^, ^3^, ^4^, ^5^, ^6^ Together, these studies demonstrated that elevated expression of many atrogenes associated with the ubiquitin proteasome pathway occurs very early, but their expression diminishes with time. Consequently, widely recognized anabolic agents may be ineffective because their targets are not consistently contributing to muscle mass loss following severe SCI. To obtain a more comprehensive understanding of the transcriptional changes that underlie the progression of SCI‐induced muscle wasting, we utilized a clinically relevant severe contusion SCI rat model that has been developed by our lab^7^ and exhibits the hallmark SCI‐induced muscle wasting phenotype in soleus muscles, including severe muscle atrophy, the predominant slow‐to‐fast muscle fibre‐type shifts, along with decreased force‐generating capacity. The soleus serves as a robust model for atrophy across multiple modes of disuse, and it also mirrors the muscle responses found in individuals with SCI (reviewed in [1]). The goal of this study was two‐fold: (1) to determine correlations between progressive pathologic features of SCI‐induced muscle wasting and transcriptional signatures and (2) to identify potential therapeutic window(s) that can target transcriptional changes in SCI that occur prior to overt muscle pathology.

## Methods

### Animals

Animal procedures conformed to the ILAR Guide to Care and Use of Experimental Animals and were approved by the Institutional Animal Care and Use Committee at the Malcolm Randall VA Medical Center. The soleus muscles assessed in this study were obtained from a larger experiment that characterized the time‐course changes of muscle,^8^ bone^9^ and locomotor function in a male rat severe contusion SCI model. Methods for surgery, post‐operative care and tissue harvest have been previously reported and are outlined in the supporting information.

### RNA extraction and RT‐qPCR

Three to five animals per group per timepoint were used for RNA‐sequencing and gene expression analysis. Total RNA was extracted from the left soleus using TriZol® Reagent (Life Technologies, 15596018). Agarose gel electrophoresis was performed to assess RNA integrity and was further quantified using a NanoDrop 2000 spectrophotometer (Thermo Fisher Scientific, ND‐2000). Recombinant DNAse I, RNAse‐free (Thermo Scientific, EN0525), was used to remove contaminating DNA. Purified RNA (500 ng) was used to generate cDNA using the High‐Capacity cDNA Reverse Transcription Kit (Applied Biosystems, 4368814). Duplicates of cDNA samples were amplified on the QuantStudio™ 3 Real‐Time PCR System using the PowerUp™ SYBR™ Green Master Mix (Applied Biosystems, A25742). These methods were performed based on the manufacturer's instructions. Gene expression was normalized to *Rpl19*, and means from age‐/sex‐matched animals that received SHAM surgery (T_9_ laminectomy) were used to determine fold change in genes of interest. *Table* *S1* lists the oligonucleotide primers used for *Rpl19* and myosin heavy chain (MHC), as previously described,^10^ along with acetylcholine receptor subunit expression.

### Bulk muscle RNA‐sequencing

RNA‐sequencing library construction and next‐generation sequencing were performed by the University of Florida Interdisciplinary Center for Biotechnology Research Gene Expression Core (RRID:SCR_019145) and the NextGen Sequencing Core (RRID:SCR_019152). Purified RNA from left soleus muscles was measured by the QUBIT fluorescent method (Invitrogen) and an Agilent Bioanalyzer. High‐quality total RNA (500 ng) with RNA integrity values above 6.9 was used for library construction using the reagents provided in the NEBNext Poly(A) mRNA Magnetic Isolation Module (New England Biolabs, E7490) and the NEBNext Ultra II Directional RNA Library Prep Kit (New England Biolabs, E7760), per the manufacturer's instructions. After magnetic mRNA isolation, the poly(A)‐enriched RNA was fragmented in NEBNext First Strand Synthesis Buffer via incubation at 94°C for the desired time. This step was followed by first‐strand cDNA synthesis using reverse transcriptase and a random hexamer primer. Synthesis of cDNA was performed using the second‐strand master mix provided in the kit, followed by end‐repair and dA‐tailing. At this point, Illumina adaptors were ligated to the sample. Finally, the library was amplified, followed by purification with AMPure beads (Beckman Coulter, A63881). The library size and mass were assessed by analysis in the Agilent TapeStation using a DNA5000 screen tape. A 200–1000 broad library peak is observed, with the highest peak at ~500 bp. Quantitative PCR was used to validate the library's functionality using the KAPA library quantification kit (Kapa Biosystems, KK4824) and monitoring on the BioRad CFX 96 real‐time PCR system. Eighteen barcoded samples were pooled equimolarly for simultaneous sequencing. The Illumina HiSeq 3000 was used to sequence the libraries for 2 × 100 cycles.

The quality of the RNA‐sequencing data was first evaluated using FastQC^11^ before further downstream analysis. Low‐quality sequences were trimmed, and poor‐quality reads were removed using Trimmomatic.^12^ The Star Aligner^13^ was used to map high‐quality single‐end reads to the genome, Rnor_6.0, downloaded from Ensembl. Gene expression was obtained using RSEM.^14^ The expected read counts and fragments per kilobase of transcript per million mapped reads (FPKM) were extracted for further analysis. The estimated read counts (*Table* *S2*) were used as input for edgeR^15^ to perform differential gene expression (DE) analysis. Generalized linear regression models were built to identify DE genes for comparisons between different treatment groups, where the respective control from the treatment groups was removed, thus eliminating the time‐course effect in SHAMs. The thresholds for calling DE genes were set at false discovery rate (FDR) 0.05 and a fold change of >2. Before DE analysis, principal component analysis (PCA) was performed to identify outlier samples. Outlier samples from 1 month SHAM (*n* = 1) and 1 week SCI (*n* = 1) were removed from the DE analysis.

### Muscle histology and morphology

Six to seven animals per group per timepoint were used for muscle histological analysis, including all animals that were used for RNA‐sequencing. Cryosections (10 μm) were generated from the mid‐belly of the right soleus. Slides were fixed in 4% paraformaldehyde for 10 min, rinsed, air dried and then stained for haematoxylin and eosin (H&E) or picrosirius red (PSR) at room temperature. PSR staining and analysis were performed as described previously.^16^ Briefly, slides were incubated in 0.1% (wt/vol) sirius red (Sigma‐Aldrich, 365548) dissolved in saturated picric acid (Ricca Chemical Company, 5860‐1) for 1 h and then washed in 0.5% acetic acid. Lastly, H&E‐ and PSR‐stained sections were dehydrated in ethanol, cleared with CitriSolv (Decon Laboratories) and mounted with Cytoseal 60 (Fisher Scientific). Three to five non‐overlapping 10× brightfield images from a PSR‐stained muscle section were captured using a Leica DMR (Leica) microscope, avoiding the epimysium. The number of red‐stained pixels was quantified using ImageJ by blinded investigators.

Immunofluorescence staining was performed for muscle fibre‐type and size analyses, along with visualization of the neuromuscular junction (NMJ). Sections were washed with phosphate‐buffered saline (PBS), blocked in 5% bovine serum albumin for 1 h at room temperature and then incubated with a cocktail of primary antibodies in a humidified chamber at 4°C overnight. After washing with PBS, sections were incubated with secondary antibodies for 1 h at room temperature in the dark and then washed again in PBS. Slides were mounted using Vectashield (Vector Laboratories). Negative control sections incubated only with secondary antibodies were included in all immunostaining analyses to avoid artefactual quantification of damaged fibres.^17^ The antibodies used are listed in *Table* *S3*. Three to four non‐overlapping 10× images (>400 fibres per animal) were captured using epifluorescence Leica DMR or confocal Leica DMI8 microscopes. Soleus muscle fibre cross‐sectional area (CSA) and fibre‐type distribution (*Figure* *S1*) were quantified using SMASH.^18^

### Hydroxyproline assay

A hydroxyproline assay was performed to quantify collagen content in soleus muscles, as described in previous studies.^16^ Muscle samples were hydrolysed overnight in 0.5 mL of 6 M hydrochloric acid at 105°C. Ten microlitres of hydrolysate was mixed with 150 μL of isopropanol, followed by 75 μL of 1.4% chloramine‐T (Sigma, St. Louis, Missouri) in citrate buffer, and oxidized at room temperature for 10 min. The samples were then mixed with 1 mL of a 3:13 solution of Ehrlich reagent (1.5 g of 4‐(dimethylamino)benzaldehyde [Sigma], 5 mL of ethanol and 337 μL of sulfuric acid) to isopropanol and incubated for 45 min at 55°C. Quantification was determined by extinction measurements of the resulting solution at 558 nm, based on an included standard curve (0–2000 μM, trans‐4‐hydroxy‐l‐proline; Sigma). Results are reported as micrograms of hydroxyproline per milligram of tissue wet weight.

### Statistical analysis

Statistical analysis was performed with GraphPad Prism 9.3.1 (GraphPad Software, Inc., CA, USA). Body weight and Basso–Beattie–Bresnahan (BBB) locomotor scores were analysed by mixed‐model repeated‐measures analysis of variance (ANOVA) along with Tukey's post hoc analysis. A two‐tailed unpaired Student's *t*‐test was used to compare SHAM and SCI at different timepoints, while a two‐way ANOVA was used to test within‐group differences across timepoints along with Bonferroni's or Tukey's post hoc analysis. Pearson's and Spearman's rank correlation coefficients were performed to analyse the relationship between locomotor function and soleus mass in each hindlimb of SCI animals, and the slope was determined by linear regression. In addition, R packages ggbiplot was used to perform PCA as described,^19^ clusterProfiler^20^ was used to perform gene ontology (GO) analysis and pheatmap^21^ was used to generate dendrograms and heatmaps. Dendrograms were generated using the *correlation* command, and the Benjamini–Hochberg adjusted *P*‐value was considered in GO analysis. The Venn diagram was generated by the Jvenn tool.^22^

## Results

### Persistent locomotor dysfunction and muscle atrophy begin early after spinal cord injury

**Figure 1:**
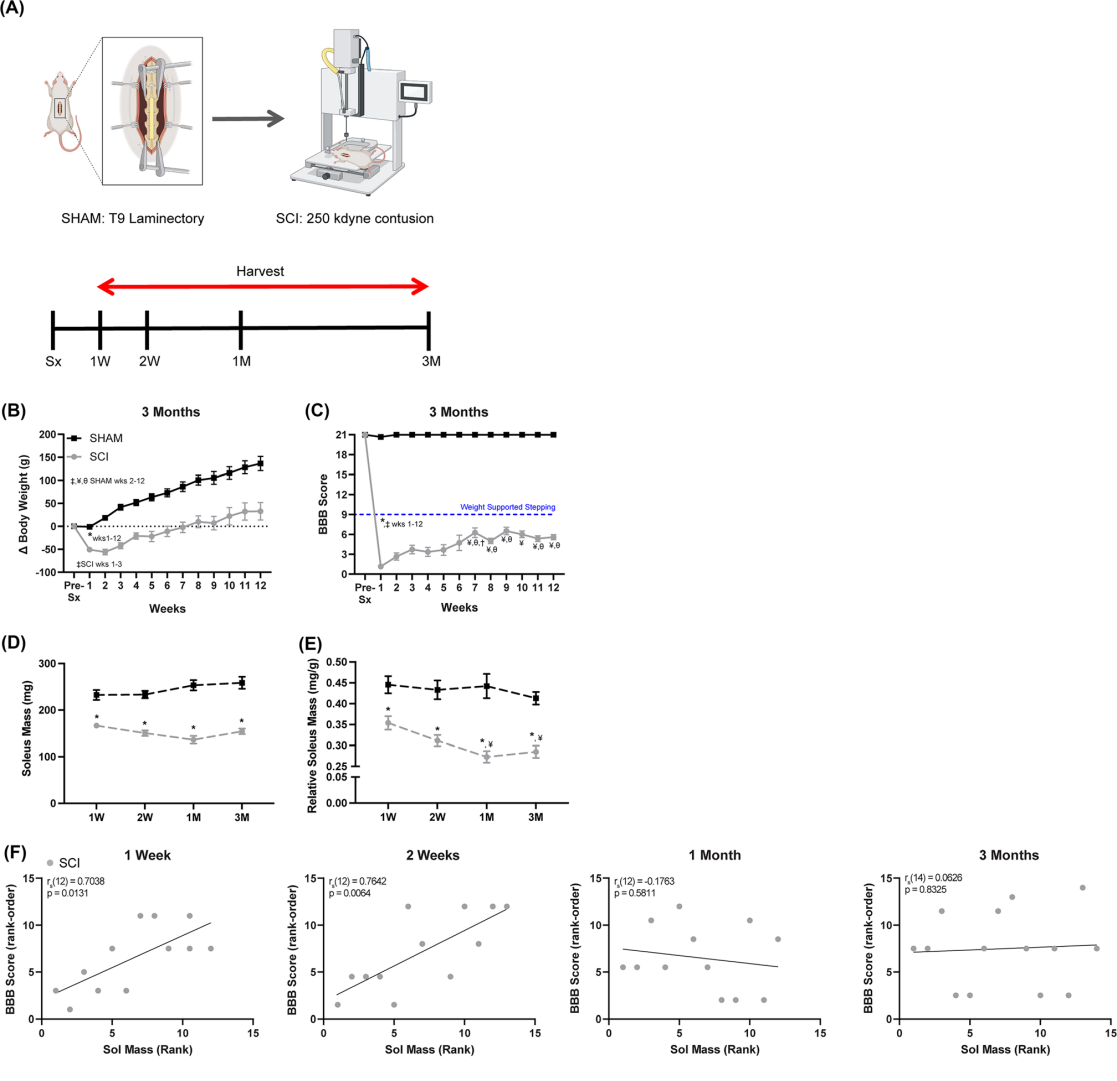
Decreased body weight, locomotor function and soleus mass from acute to chronic SCI. (A) Scheme showing SHAM and SCI experimental timepoints where soleus muscles from male rats were harvested at 1 week (1 W), 2 weeks (2 W), 1 month (1 M) and 3 months (3 M) post‐injury. (B) SCI animals from the 3 M group had lower body weight from Weeks 1–3 post‐injury when compared with pre‐surgery (pre‐Sx) but gained body weight from Week 7 onwards. (C) The hindlimb locomotor function of SCI animals remained below the ability to support the hindlimbs in stance or to perform weight‐supported stepping levels (Basso–Beattie–Bresnahan [BBB] < 9 throughout). (D, E) The absolute and relative soleus masses of SCI animals were lower than SHAMs at all post‐injury timepoints. (F) A positive linear relationship existed between BBB score (rank order) and soleus mass (rank order) when the right and left hindlimbs of SCI animals were assessed separately at 1 W (95% confidence interval [CI]: 0.1994 to 0.9133) and 2 W (95% CI: 0.3219 to 0.9327) post‐injury; however, this correlation disappeared at the 1 M (95% CI: −0.6915 to 0.4577) and 3 M timepoints (95% CI: −0.4973 to 0.5857). The data presented in (B)–(E) are the mean ± SEM. *P < 0.05 between SHAM and SCI, *P* < 0.05: ǂ versus pre‐surgery, ¥ versus 1 W, θ versus 2 W and † versus 1 M within group (Tukey's and Bonferroni's post hoc analyses), *n* = 6 SHAM, *N* = 6–7 SCI. *Source*: Figure (A) was generated in BioRender.

We have previously reported pooled observations of the changes in BBB scores, body mass and muscle mass from a rat severe contusion SCI model at 2 weeks, 1 month and 3 months post‐injury.^8^, ^9^ Herein, we added the 1 week timepoint to capture earlier changes in gene expression and to determine their temporal relationship with muscle atrophy (*Figure* *1* *A*). Like previous reports, SCI animals exhibited lower body weights than SHAMs throughout the study (*Figures* *1* *B* and *S2A*). Moreover, SCI animals did not regain the ability to support the hindlimbs in stance or perform hindlimb weight‐supported stepping (BBB < 9 throughout) (*Figures* *1* *C* and *S2B*) and had lower absolute and relative soleus muscle mass compared with SHAM (*Figure* *1* *D,E*) at all post‐surgery timepoints.^8^, ^9^ Spearman's rank and correlation coefficients indicated positive associations between locomotor function (BBB rank‐order and raw values) and soleus mass only at 1 week and 2 weeks in SCI animals (*Figures* *1* *F* and *S3*). This is consistent with previous studies indicating that locomotor function and muscle CSA were positively correlated prior to the 1 month timepoint in rats receiving severe contusion SCI^23^ and expands upon these data by demonstrating that no association exists between soleus mass and BBB scores thereafter. Taken together, locomotor dysfunction and soleus muscle atrophy persisted throughout the acute to chronic post‐SCI phase.

### Transcriptomics analysis reveals enriched gene signatures for extracellular matrix and skeletal muscle contractile protein remodelling after spinal cord injury

**Figure 2:**
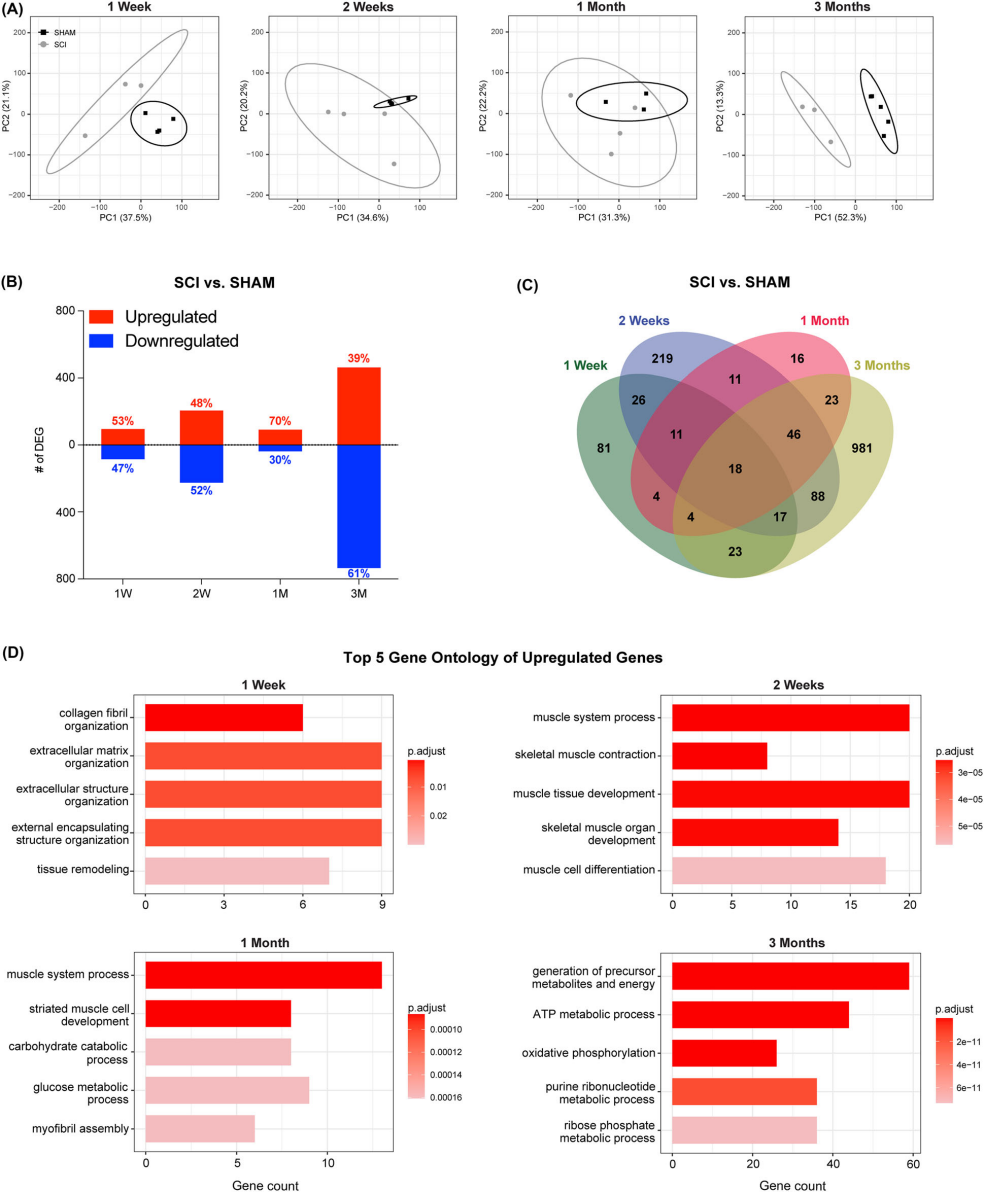
Transcriptomics analysis showed predominant signatures in the remodelling of the extracellular matrix and skeletal muscle. (A) The principal component analysis (PCA) plot showed differences at the 1 week (1 W) and 3 months (3 M) timepoints. At the 1 month (1 M) timepoint, there was high variability and significant overlap of SHAM and SCI ellipses (95% confidence interval [CI]). (B) The number of differentially expressed genes (DEGs) was highest at the 3 M timepoint. (C) Venn diagram showing number of common DEGs at all timepoints, where 18 significantly changed genes were common to all timepoints throughout the study. The highest number of exclusive genes were in the 3 M timepoint. (D) The top 5 enriched gene ontology biological process terms for upregulated DEGs indicate remodelling of the extracellular matrix at 1 W, muscle fibres at 2 weeks (2 W) and 1 M and changes in fuel utilization at 3 M.

To delineate the molecular changes that accompany muscle wasting after SCI, we performed bulk RNA‐sequencing analysis of soleus muscles from SCI and SHAM animals at 1 week, 2 weeks, 1 month and 3 months post‐surgery. Soleus muscles were utilized as they are important postural muscles that undergo severe atrophy after SCI and that develop a phenotypic slow‐to‐fast fibre‐type shift within the time course of our study.^7^ PCA performed on the RNA‐sequencing datasets revealed that SHAM and SCI groups differed at 1 week and 3 months timepoints but overlapped at the 2 weeks and 1 month timepoints (*Figure* *2* *A*). These data supported that transcriptional differences were most prominent early and late in the responses to SCI, whereas intermediate timepoints may have exhibited changes due to translational or post‐translational processes. To account for deviations across timepoints within the SHAM group (18–32%), indicative of the variance in outbred Sprague–Dawley rats, we compared differentially expressed genes (DEGs) between SCI and SHAM at each timepoint. In total, there were 184 DEGs at 1 week, 436 DEGs at 2 weeks and 133 DEGs at 1 month, and surprisingly, the most abundant change was captured at 3 months (1200 DEGs) due to many more genes being downregulated in SCI versus SHAM at this timepoint (*Figure* *2* *B*). Comparisons of all DEGs revealed 18 genes that were common to all timepoints (Figure 2C and Table S4). These gene expression patterns demonstrate a progressive and dynamic transcriptional landscape at least through 3 months post‐SCI.

GO analysis revealed both transient and persistent biological processes from upregulated and downregulated DEGs. First, genes associated with extracellular matrix (ECM) signatures were enriched within the upregulated DEGs at 1 week but were later enriched in the downregulated DEGs at 2 weeks and 3 months (Figure 2D and Table S5), suggesting that transcriptional ECM remodelling likely occurred early and in a transient manner after SCI. Second, GO terms associated with muscle development, differentiation and contraction were enriched within the upregulated DEGs at 2 weeks and 1 month and subsequently in the downregulated DEGs at 3 months, an indication that transcriptional changes associated with skeletal muscle fibre remodelling began relatively early post‐SCI. Third, the emergence of metabolic GO terms occurred at 1 month and was more prominent at 3 months, suggesting that shifts in energy utilization may have occurred after the remodelling of the contractile apparatus. Collectively, the transcriptional profile pointed to three major targets of remodelling: the ECM, muscle fibres and metabolism. Because many therapeutic strategies attempt to counter the loss of muscle mass and function, the following sections delve further into the identity of pathways and the time course of factors that may influence muscle atrophy, muscle fibre remodelling and muscle fibrosis. Because our study design eliminated the availability of fresh tissue after the acquisition of RNA‐sequencing data, we did not pursue metabolism pathways.

### Muscle atrophy is driven by pro‐catabolic and anti‐growth genes after spinal cord injury

**Figure 3:**
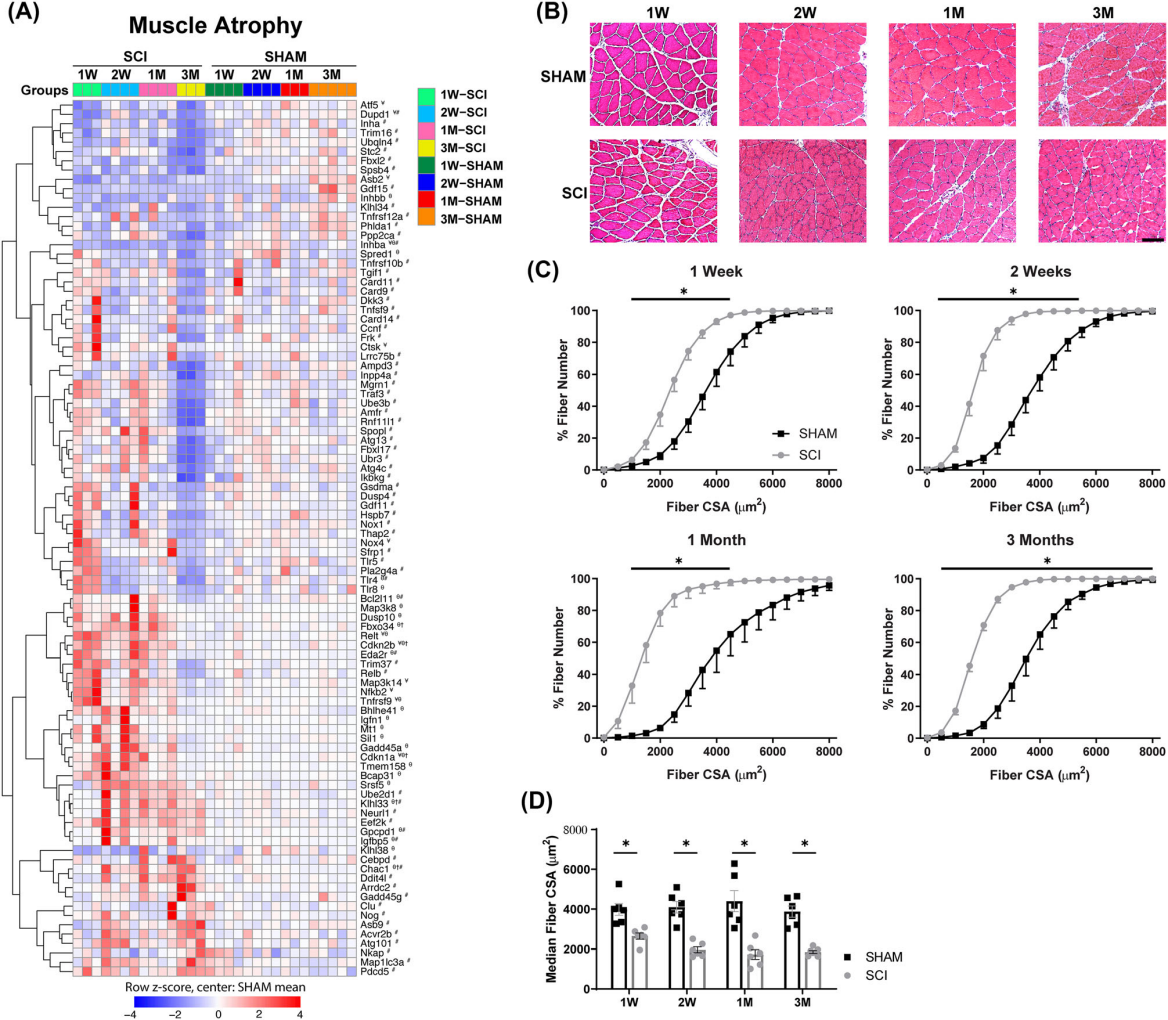
Persistent muscle atrophy from acute to chronic SCI. (A) DEGs relevant to the molecular mechanisms of muscle atrophy. (B) Representative images of haematoxylin and eosin‐stained soleus muscles from SHAM and SCI at 1 week (1 W), 2 weeks (2 W), 1 month (1 M) and 3 months (3 M). Scale bar: 100 μm for all images. (C) The per cent cumulative distribution of muscle fibre areas from SHAM and SCI animals at 1 W, 2 W, 1 M and 3 M post‐surgery showed a leftward shift in SCI muscle fibre area at all timepoints when compared with SHAM. (D) The median fibre area significantly decreased in the SCI soleus but remained persistent throughout the study. The data presented in (A) are the row z‐score, where 0 represents the SHAM mean. DEGs are indicated as SCI versus SHAM: ¥ at 1 W, θ at 2 W, † at 1 M and # at 3 M. The data presented in (C) and (D) are the mean ± SEM. *P < 0.05 between SHAM and SCI, with the bar in (C) representing significant differences in the range of fibre areas.

To identify transcriptional changes in known pathways that contribute to the loss of muscle mass in other atrophy models,^24^, ^25^, ^26^ DEGs associated with muscle atrophy were interrogated within the RNA‐sequencing dataset that ranged from 1 week to 3 months post‐SCI. Cell senescence markers, the cyclin‐dependent kinase inhibitors *Cdkn1a* (p21) and *Cdkn2b* (p15^INK4b^), were continually upregulated from 1 week to 1 month post‐SCI, along with *Tmem158* at 2 weeks (*Figure* *3* *A*). Genes that regulate the nuclear factor kappa B (NF‐κB) pathway, including *Map3k14*, *Nfkb2*, *Tnfrsf9*, *Relt*, *Map3k8* and *Eda2r*, were upregulated in SCI muscles at 1 week and/or 2 weeks, along with *Nkap* at 3 months. Genes associated with proteasome activity that were upregulated by 2 weeks post‐SCI included *Klhl33* and *Fbxo34*, along with *Neurl1*, *Asb9*, *Ube2d1* and *Cebpd* at 3 months post‐SCI. Additionally, genes associated with the endoplasmic reticulum (ER) stress/unfolded protein response (UPR) pathway were upregulated at/from 2 weeks post‐SCI, including *Bcap31*, *Sil1* and *Chac1*, along with *Clu* at 3 months post‐SCI. Apoptotic markers that were upregulated in SCI muscles include *Bcl2l11* at 2 weeks but downregulated at 3 months, while *Pdcd5* was upregulated only at 3 months. Alterations in genes associated with autophagic activity occurred 3 months post‐SCI, as demonstrated by the upregulation of *Map1lc3a* and *Atg101*, an indication of increased autophagosome formation. Hallmark markers of denervation‐induced muscle atrophy were also upregulated 2 weeks and/or 3 months post‐SCI, including *Gadd45a*, *Gpcpd1*, *Arrdc2* and *Gadd45g*. Taken together, early catabolic gene signals post‐SCI are related to cell senescence, the NF‐κB pathway, the proteasome and the ER stress/UPR systems, whereas those associated with autophagy appear later.

The transforming growth factor beta (TGFβ) superfamily plays a major role in the coordination of muscle anabolism and catabolism. For instance, the bone morphogenic protein (BMP) subfamily promotes muscle growth, while other TGFβ ligands such as activins and myostatin suppress muscle growth.^23^, ^27^ At 3 months post‐SCI, noggin (*Nog*), a BMP antagonist, and the myostatin/activin receptor (*Acvr2b*) were upregulated, suggesting that alterations in TGFβ signalling occur at a later timepoint after SCI and appear to be predominantly anti‐growth. Indeed, genes that are associated with decreases in protein syntheses such as *Igfn1* and *Eef2k* were upregulated at 2 weeks and 3 months post‐SCI, respectively. Major drivers of protein synthesis in the skeletal muscle include the insulin‐like growth factor‐I (IGF‐I)/Akt/mechanistic target of rapamycin (mTOR) and p38/mitogen‐activated protein kinase (MAPK) signalling. Genes that blunt the IGF‐I/Akt/mTOR signalling cascade, including *Ctsk*, *Mt1*, *Igfbp5* and *Ddit4l*, were upregulated 1 week, 2 weeks and/or 3 months post‐SCI. In the same manner, *Dusp10*, a negative regulator of the p38/MAPK signalling cascade, was upregulated 2 weeks post‐SCI. Ultimately, we suspect that the combination of increased expression of catabolic genes combined with negative regulators of muscle growth contributed to the persistent decrease in muscle fibre area from 1 week to 3 months post‐SCI (*Figure* *3* *B–D*).

### Transcriptional and translational evidence of muscle fibre‐type shifts after spinal cord injury

**Figure 4:**
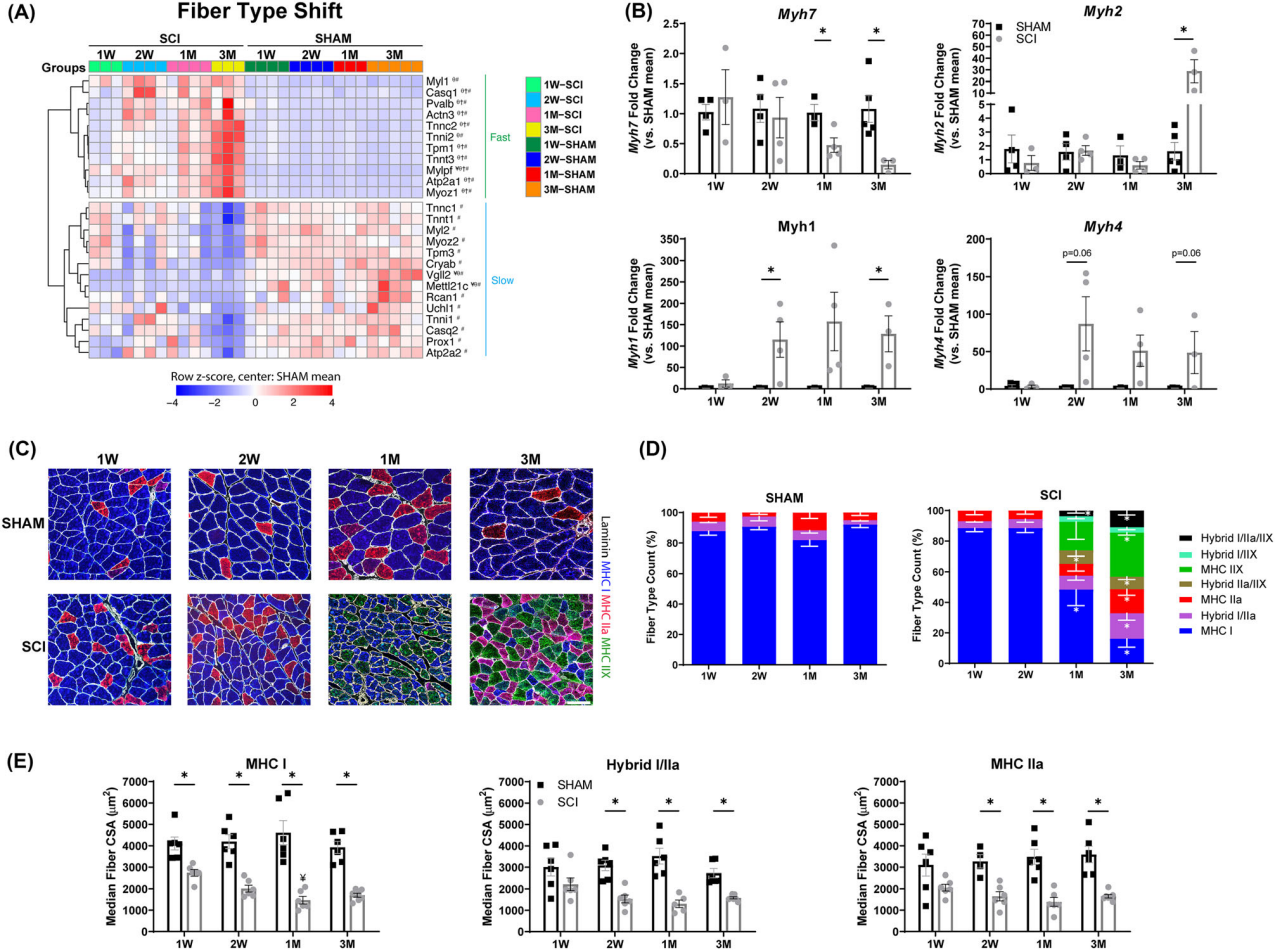
Fibre‐type shifts in soleus muscle from chronic SCI. (A) RNA‐sequencing analysis revealed a shift in DEGs representing the slow and fast fibre programmes in SCI muscles, where downregulated slow genes and upregulated fast genes are observed in SCI animals. (B) Gene expression analysis (RT‐qPCR) of the soleus muscle validated fibre‐type shifts, where *Myh7* was downregulated while *Myh2* and *Myh1* were upregulated in SCI animals at various timepoints. (C) Representative soleus muscle fibre‐type images stained with MHC I (blue), MHC IIa (red), MHC IIX (green) and laminin (white). Scale bar: 100 μm for all images. (D) SCI muscle fibre‐type distribution showed a progressive shift from slow‐to‐fast fibres, with a decrease in slow fibres and an increase in fast fibres along with increased hybrid fibres. (E) The median fibre area of MHC I is significantly smaller in the SCI soleus at all timepoints, and the fibre areas of hybrid I/IIa and MHC IIa are significantly smaller from 2 weeks (2 W) post‐injury onwards. The data presented in (A) are the row z‐score, where 0 represents the SHAM mean. DEGs are indicated as SCI versus SHAM: ¥ at 1 week (1 W), θ at 2 W, † at 1 month (1 M) and # at 3 months (3 M). The data presented in (B), (D) and (E) are the mean ± SEM. *P < 0.05 between SHAM and SCI, *P* < 0.05: ¥ versus 1 W within group (Bonferroni's post hoc analysis).

The rat soleus is typically composed of more than 90% slow‐oxidative type I fibres, ~5% fast‐oxidative type IIa fibres and a small percentage of hybrid I/IIa fibres.^28^ We previously reported slow‐to‐fast fibre‐type transitions in male rats 2 months after severe contusion SCI,^7^ replicating clinical findings in SCI patients.^29^ Thus, we expected that the SCI soleus would undergo a transition from type I to type IIa fibres. First, we analysed the changes in DEGs for distinct muscle fibre‐type profiles.^30^, ^31^, ^32^ In the fast fibre programme, upregulation of the fast myosin light chain (*Mylpf*) was observed at all timepoints, while *Actn3*, *Pvalb*, *Atp2a1*, *Tnnc2*, *Tnnt3*, *Myoz1*, *Tpm1*, *Casq1*, *Tnni2* and *Myl1* were upregulated at 2 weeks or later timepoints post‐SCI (*Figure* *4* *A*). In the slow fibre programme, *Vgll2* and *Mettl21c* were downregulated 1 week, 2 weeks and 3 months post‐SCI, along with *Rcan1*, *Uchl1*, *Tpm3*, *Casq2*, *Prox1*, *Myoz2*, *Cryab*, *Myl2*, *Tnnc1*, *Atp2a2*, *Tnni1* and *Tnnt1* at 3 months. To validate the transcriptional shifts in fibre‐type profiles after SCI, RT‐qPCR was performed, and *Myh7* (type I) was progressively downregulated from 1 to 3 months post‐SCI, while *Myh2* (type IIa) was upregulated by more than 20‐fold at 3 months versus SHAMs (*Figure* *4* *B*). Additionally, *Myh1* (type IIX) was also upregulated at 2 weeks and 3 months, along with non‐significant trends for *Myh4* (type IIb) in the SCI soleus. The slow‐to‐fast transcriptional changes were substantiated by immunostaining of MHC proteins (*Figure* *4* *C*). Major changes emerged at 1 and 3 months post‐SCI, where the presence of IIX fibres and an increased proportion of hybrid fibres were evident (*Figures* *4* *D* and *S4A*). Undoubtedly, significant remodelling of the cytoskeleton accompanies fibre‐type turnover in SCI muscles (*Figure* *S4B*). Not only was the proportion of MHC I fibres lower in SCI, but the fibre area was smaller throughout when compared with SHAMs (*Figure* *4* *E*). Interestingly, hybrid I/IIa and MHC IIa fibre atrophy was not significant in the soleus until 2 weeks post‐SCI, supporting that type I fibres are more susceptible to atrophy than type II fibres. Collectively, we observed evidence of slow‐to‐fast fibre transitions by gene expression and by immunofluorescence analysis in our severe contusion SCI model.

### Signs of neuromuscular junction remodelling are evident after spinal cord injury

**Figure 5:**
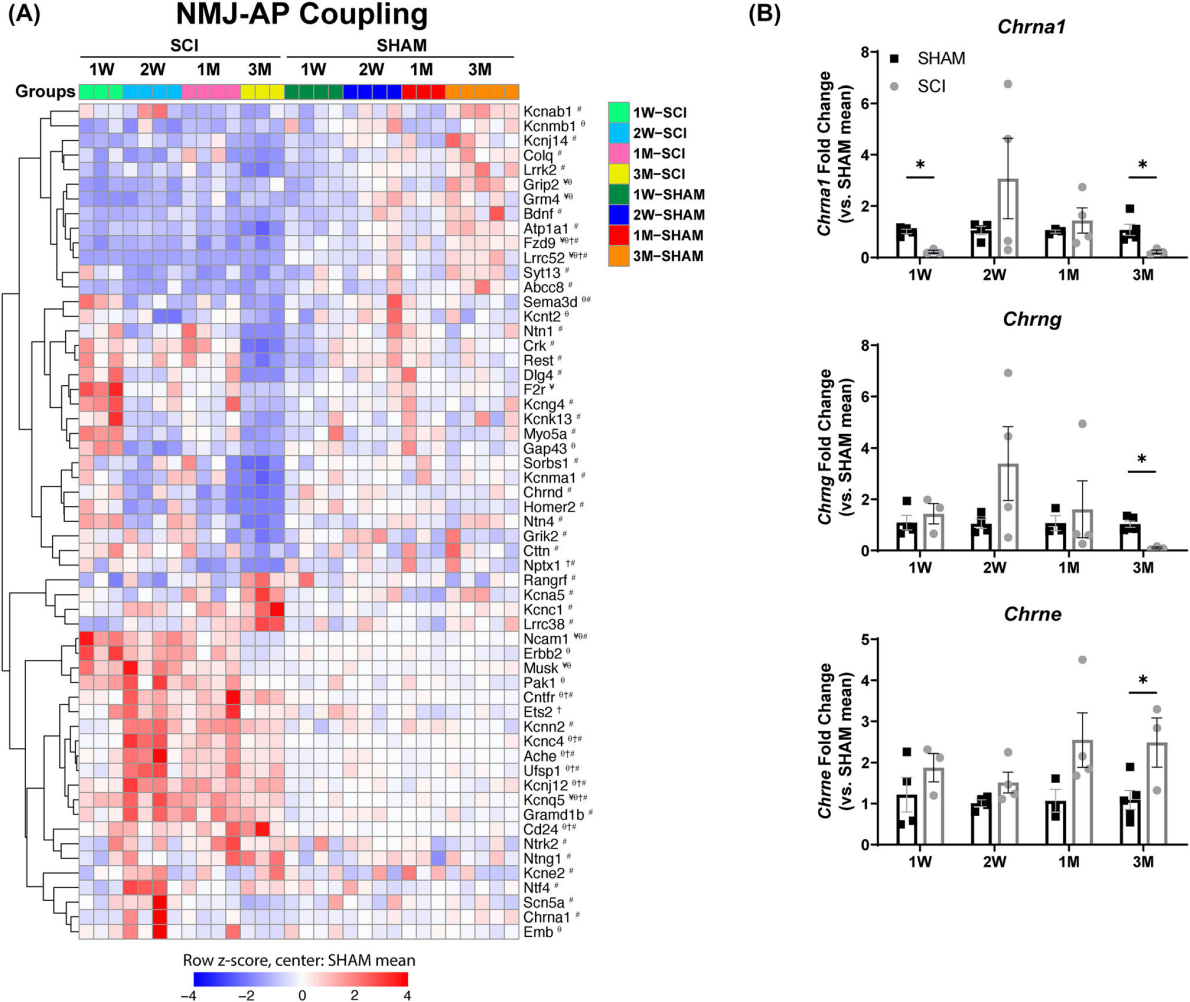
Transcriptomic changes that regulate skeletal muscle excitation and relaxation in the SCI soleus. (A) DEGs that depict molecular mechanisms in the regulation of endplate and action potential (AP) across individual SCI and SHAM animals. (B) Validation of RNA‐sequencing results with RT‐qPCR of the soleus muscle displayed significant downregulation in *Chrna1* and *Chrng* but significant upregulation in *Chrne* at 3 months (3 M) in SCI animals. The data presented in (A) are the row z‐score, where 0 represents the SHAM mean. DEGs are indicated as SCI versus SHAM: ¥ at 1 week (1 W), θ at 2 weeks (2 W), † at 1 month (1 M) and # at 3 M. The data presented in (B) are the mean ± SEM. *P < 0.05 between SHAM and SCI. NMJ, neuromuscular junction.

Axonal degeneration due to SCI may produce cyclic motor neuron denervation, exacerbating the loss of muscle mass and strength.^33^ Thus, we evaluated genes that are known to regulate skeletal muscle excitation in other rodent models to determine if their expression was altered in response to SCI. First, we analysed the genes associated with NMJ stability. Genes involved in NMJ synapse formation and acetylcholine receptor clustering were upregulated between 1 week and 3 months post‐SCI: *F2r*, *Musk*, *Pak1*, *Erbb2*, *Cd24*, *Ets2* and *Gramd1b* (*Figure* *5* *A*). Interestingly, *Ufsp1*, a negative regulator of acetylcholine clustering and NMJ formation, was also upregulated from 2 weeks to 3 months. In comparison, key genes regulating the maintenance of the postsynaptic apparatus were downregulated only at 3 months post‐SCI: *Colq*, *Chrna1*, *Chrnd*, *Dlg4*, *Myo5a* and *Crk*. To determine if a switch from adult (*Chrne*) to foetal (*Chrng*) acetylcholine subunits occurred in SCI muscles, RT‐qPCR was performed using primers specific to each subunit. Consistent with RNA‐sequencing data, *Chrna1* was downregulated 3 months post‐SCI (*Figure* *5* *B*). Importantly, downregulation of *Chrng* and upregulation of *Chrne* at 3 months suggest that early disruption of the NMJ, including the acetylcholine receptor (*Figure* *S5*), may have resolved. Next, we asked if the motor neuron was affected by muscle atrophy and NMJ destabilization, given previous reports on the regulation of muscle on neurite outgrowth and a shift in motor neuron identity.^34^, ^35^ Indeed, transcription of genes known to regulate neurotrophic factors that promote motor neuron survival or growth, *Ncam1*, *Emb*, *Cntfr*, *Ntrk2*, *Ntng1* and *Ntf4*, was upregulated at various timepoints between 1 week and 3 months post‐SCI (*Figure* *5* *A*). Action potentials on the myofiber are regulated by voltage‐gated sodium channels. At 3 months post‐SCI, *Scn5a* (Nav 1.5) was downregulated while *Rangrf* (MOG 1), known to enhance the activity of Nav 1.5, was upregulated. Additionally, acetylcholinesterase (*Ache*), which hydrolyses the remaining acetylcholine in the synaptic cleft, was continuously upregulated from 2 weeks to 3 months post‐SCI. While further testing of protein levels and muscle excitability was not assessed, our analysis is consistent with previous studies demonstrating that SCI alters gene expression patterns in a manner that can influence NMJ remodelling and/or excitability.

### Transcription of fibrosis genes is early but transient in spinal cord injury soleus

**Figure 6:**
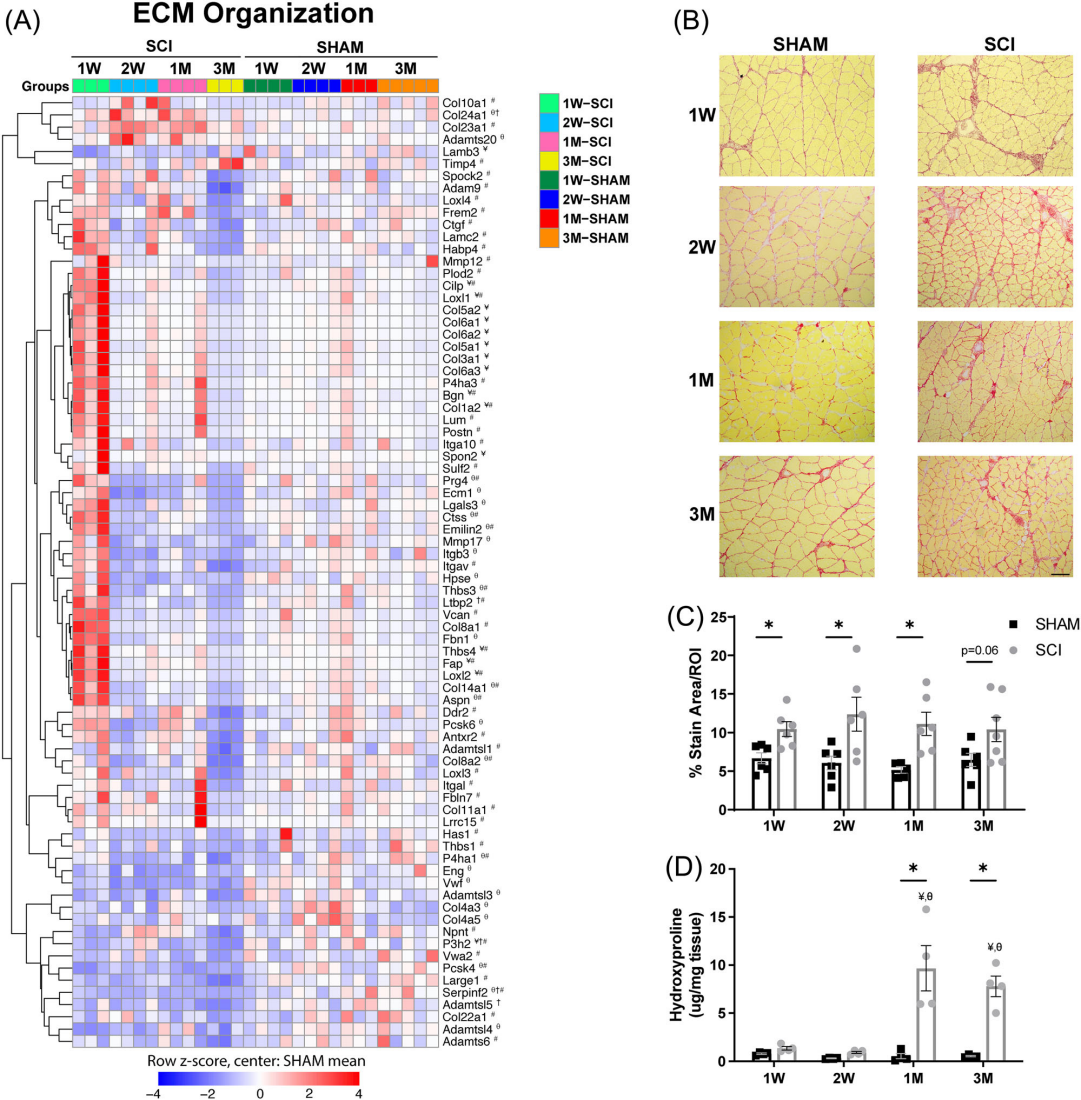
Rapid muscle fibrosis in SCI muscles. (A) DEGs that regulate extracellular matrix (ECM) organization. (B) Representative images of soleus muscle from SHAM and SCI stained with picrosirius red at 1 week (1 W), 2 weeks (2 W), 1 month (1 M) and 3 months (3 M). Scale bar: 100 μm for all images. (C) The per cent ECM area (red) in SCI soleus was significantly higher at the 1 W, 2 W and 1 M timepoints. (D) Total collagen content in SCI soleus was significantly higher at the 1 M and 3 M timepoints. The data presented in (A) are the row z‐score, where 0 represents the SHAM mean. DEGs are indicated as SCI versus SHAM: ¥ at 1 W, θ at 2 W, † at 1 M and # at 3 M. The data presented in (C) and (D) are the mean ± SEM. *P < 0.05 between SHAM and SCI, *P* < 0.05: ¥ versus 1 W and θ versus 2 W, within group (Tukey's post hoc analysis). ROI, region of interest.

Persons with chronic SCI exhibit muscle fibrosis.^6^ Thus, we investigated the temporal pattern of transcripts involved in muscle fibrosis and found that many ECM genes were upregulated at 1 week but downregulated thereafter. Upregulated collagen genes included *Col1a2*, *Col3a1*, *Col5a1*, *Col5a2*, *Col6a1*, *Col6a2* and *Col6a3* at 1 week, *Col24a1* at 2 weeks and 1 month post‐SCI and *Col23a1* at 3 months post‐SCI (*Figure* *6* *A*). Collagen crosslinkers *Loxl1* and *Loxl2* were also upregulated 1 week post‐SCI but were subsequently downregulated at 3 months. Several genes that regulate proteoglycans were also upregulated 1 week post‐SCI, including *Spon2*, *Thbs4* and *Bgn*. Similarly, other genes that are associated with ECM integrity were upregulated 1 week post‐SCI and then downregulated at 3 months, including *Cilp* and *Fap*. Interestingly, expression of many ECM genes was downregulated at 2 weeks and beyond. In contrast, *Col24a1* was still upregulated at 2 weeks and 1 month post‐SCI, and *Timp4* was upregulated at 3 months post‐SCI, potentially indicating that matrix metalloproteinase (MMP) actions necessary for ECM degradation may have been inhibited. The transient upregulation of fibrogenic genes in SCI soleus was accompanied by a higher ECM area that was evident by 1 week and persisted thereafter (*Figure* *6* *B,C*). Therefore, a temporal separation between the early transient expression of genes contributing to collagen accumulation and its stability and the persistence of fibrotic tissue that was evident early after SCI. In particular, biochemical evidence of collagen accumulation was apparent only at 1 month and 3 months post‐SCI. Altogether, these data indicate that increased ECM gene expression is an acute phenomenon that develops early after SCI in our model, but the consequences of this gene expression in terms of fibrosis are delayed and persistent.

## Discussion

**Figure 7:**
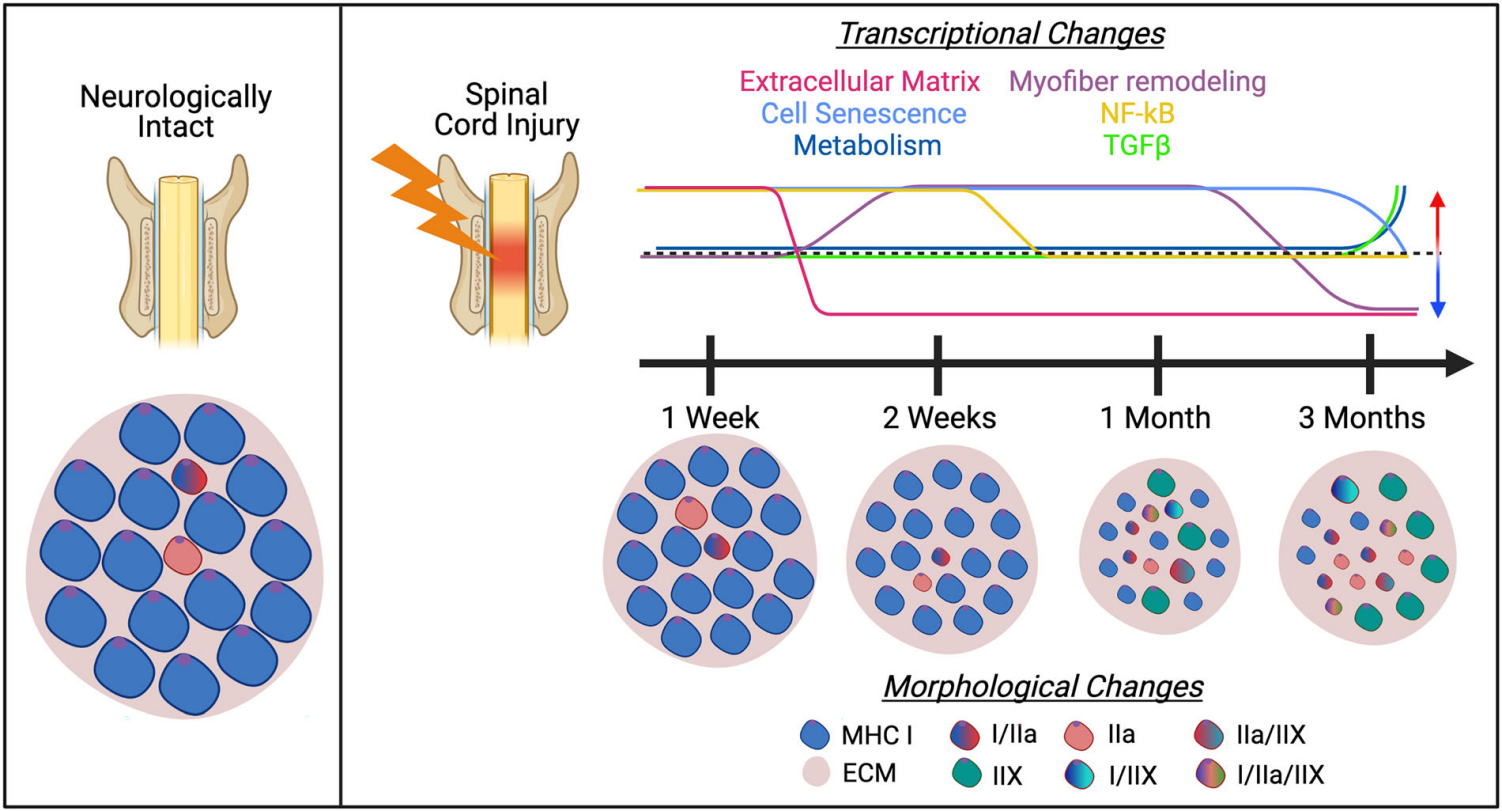
Scheme representing transcriptional and morphological time‐course changes after SCI. Genes representing extracellular matrix (ECM) remodelling are upregulated at 1 week (1 W) post‐SCI but downregulated from 2 weeks (2 W) onwards. Genes that depict the remodelling of myofiber were upregulated at 2 W and 1 month (1 M) post‐SCI and subsequently downregulated at 3 months (3 M). Atrophic signatures, including NF‐κB, were upregulated from 1 to 2 weeks post‐SCI; cell senescence markers were upregulated from 1 week to 1 month; and TGFβ was upregulated at 3 months. Slow‐to‐fast fibre‐type shifts were evident at 1 M and 3 M post‐SCI, which accompanied the upregulation of fuel utilization genes at 3 M. Overall, SCI results in a multifaceted pathology in the skeletal muscle. MHC, myosin heavy chain. *Source*: The figure was generated in BioRender.

Skeletal muscle wasting is a rapid and debilitating consequence of severe SCI. Axonal degeneration post‐SCI causes muscle paralysis and, combined with skeletal muscle unloading, exacerbates rapid fibre atrophy. The rat severe contusion SCI model exhibits all facets of the human condition, but over an accelerated time course. This has enabled examination of transcriptional changes following severe SCI that mirrored many months to years in humans. RNA‐sequencing analysis complemented with morphological assessments was performed to determine molecular pathway changes that were temporally linked with acute to chronic SCI‐induced muscle wasting and to identify potential therapeutic window(s) to target the pathways with transcriptional interventions that are independent of the later morphologic and functional consequences for the muscle tissue. We found that early and transient expression of ECM remodelling genes was accompanied by muscle fibrosis as early as 1 week after SCI. Rapid muscle fibre atrophy was also evident and coincided with a broad range of gene expression changes unique to SCI. Progressive remodelling of fibre properties grew with time and included not only changes in genes that are known to regulate contractile proteins but later changes in genes that may influence metabolism (*Figure* *7*).

Persistence of soleus muscle atrophy has been previously reported from 1 week to 2 months after severe SCI,^7^, ^23^, ^36^ a trend that we have now extended to 3 months. Even though muscle atrophy is a prominent and early feature of SCI, GO terms associated with general anabolic or catabolic processes were absent, suggesting that transcriptional changes of known atrophic processes preceded the 1 week timepoint and/or that the muscle mass loss in our study depended upon post‐transcriptional atrophic drive or involved the expression of additional atrophy genes. For instance, well‐known atrogenes such as Murf1 (*Trim63*), Atrogin1 (*Fbxo32*) and Musa1 (*Fbxo30*) were not differentially expressed in the soleus muscle of severe contusion SCI rats from 1 week onwards.^24^, ^26^ Similarly, the FOXO transcription factors, which have been shown to play a role in the regulation of Atrogin1, were not present in the DEG list throughout the study.^25^ Human studies have found upregulation of atrogene expression 2–5 days after injury,^3^, ^4^ that diminished later on,^5^, ^6^ which is consistent with our observations. Instead, we found that genes that modulate NF‐κB signalling were upregulated concurrently with muscle atrophy 1 week post‐SCI, along with genes that influence cell senescence markers (p21 and p15INK4b). NF‐κB drives the upregulation of the ubiquitin proteasome system in denervation atrophy.^37^, ^38^ In our model, any potential change in NF‐κB activity may have induced proteasome activity from 2 weeks to 3 months (*Fbxo34*, *Klhl33*, *Ube2d1*, *Neurl1*, *Cebpd* and *Asb9*), along with pro‐apoptotic markers. Thus, we propose that catabolic stimuli in SCI‐induced muscle atrophy may be distinct from other muscle wasting conditions, in agreement with a previous study.^39^ For instance, bulk RNA‐sequencing comparisons of tibialis anterior (TA) muscles from mice with skeletal muscle atrophy induced by dexamethasone, Lewis lung carcinoma (LLC) cancer cells or aging had distinct signatures, where gene set enrichment analysis (GSEA) revealed upregulation of ‘collagen metabolic process’ with dexamethasone, ‘response to lipoprotein particle’ with cachectic LLC cancer and ‘heterotypic cell–cell adhesion’ with aging.^39^ Even so, *Cdkn1a* was upregulated in all atrophy models listed in Hunt et al.^39^, as well as our SCI soleus muscle, supporting the overlap of atrophy pathways independent of cause.^24^ One consideration we did not pursue was the fibre‐type specificity of anti‐growth and pro‐catabolic gene expression changes after SCI. Distinct genes could drive type I fibre atrophy at 1 week post‐SCI (e.g., NF‐κB signalling), while other pathways may drive type IIa fibre atrophy at later timepoints. Alternatively, these pathways may contribute to myofiber remodelling and turnover as indicated by GO analysis, to accommodate the fibre‐type shift evident from 1 month post‐SCI. These questions cannot be addressed through bulk RNA‐sequencing. However, identification of the time course in which these catabolic gene targets are altered in relation to muscle atrophy provides a therapeutic window that future studies may utilize to mechanistically determine their potential role in atrophy following SCI.

The slow‐to‐fast fibre shift in SCI occurs as a continuum in both gene expression and protein levels, and it is likely accompanied by cyclic denervation–reinnervation. This is potentially attributed to increases in nodal sprouting from fast fibres in partially denervated muscles.^40^ Although we did not examine NMJ integrity longitudinally, the presence of genes that regulate neurotrophic factors throughout the timepoints raises the potential for targeting motor neuron survival by either boosting neurotrophic factors from the muscle (e.g., *Ncam1*) or repressing negative regulators of synaptic activity (e.g., *Ufsp1* and *Ache*) post‐SCI. Such strategies, combined with neuromuscular electrical stimulation^2^, ^41^ or other activity‐based physical therapy modalities, may synergize to improve muscle recovery after SCI.^42^

The fibre‐type shifts following SCI pervaded virtually all aspects of fibre properties, from contractile proteins through alterations in metabolism, and continued to progress at each timepoint. It is an open question as to how ‘fast’ the soleus might become with longer monitoring. Notably, the appearance of genes that are associated with metabolic changes followed those of the contractile apparatus. It is not clear if correction of fibre type, per se, is an important therapeutic target. However, a shift in fuel utilization can have systemic consequences for individuals, and it has been actively pursued through exercise regimens for upper extremity muscles.^43^ In our study, we did not investigate the metabolic shifts in the rat model because these appeared quite late in the time course and because we utilized tissues that prevented robust analysis of enzymatic activity. As such, a thorough examination of the metabolic adaptations within the soleus is needed to validate the functional metabolic consequences of SCI within the soleus and would benefit from a longer study duration and assessments such as glucose uptake, mitochondrial respiration, analysis of proteins involved in oxidative phosphorylation or mitochondrial markers, as well as immunohistochemistry to determine the changes in fuel utilization.

A contributing factor to muscle stiffness after SCI is extensive muscle fibrosis. The rat model exhibited a temporal separation between the transcriptional changes in ECM‐related genes, the morphological appearance of fibrosis and the biochemical index of collagen accumulation. An early phase of active ECM remodelling may have set the stage for increased and established fibrosis and suggests that anti‐fibrotic agents that target gene expression may not be effective in this pre‐clinical model unless they are introduced immediately following injury. The more static nature of the transcriptional signature for ECM at later timepoints suggests that the accumulation of fibrosis may be much slower. This could be therapeutically advantageous for anti‐fibrotic agents that prevent ECM assembly or enhance its degradation. Biochemical measurements of hydroxyproline (*Figure* *6D*) indicate that the fibrosis is persistent at later timepoints. The fact that at 1 week and 2 weeks there is increased PSR staining in SCI samples even though there is not an elevation in hydroxyproline levels suggests that the imaging analysis of PSR‐stained sections is affected by the pronounced atrophy. In other words, because the fibres are smaller, the ECM has greater representation in any given region of interest (ROI). However, by 1 month and 3 months, the collagen content increases, likely reflecting more fibrotic deposition in the muscles. Combined, these data suggest that the ability of the muscle to degrade collagen might be impaired, as opposed to a constant upregulation of collagen deposition. Whether a similar temporal pattern occurs in SCI patients has not been evaluated, even though fibrosis is a significant consequence of SCI.

Overall, these results substantiate many of the known consequences of severe SCI in rat skeletal muscles, and this study has identified potential therapeutic window(s) to target transcriptional changes that occur prior to muscle pathology in this model, which may open new possible avenues for treatments. Importantly, not only the targets and pathways but also the transient nature of their contribution to SCI‐related pathologies should be considered in therapeutic strategies.

## Funding

This work was supported by the Department of Veterans Affairs Office of Research and Development, Rehabilitation Research and Development Service Grants 1I21RX001373‐01 and PECASE B9280‐O to JFY, by the US National Institutes of Health (NIH) Grant P50 AR052646 to ERB and by resources provided by the North Florida/South Georgia Veterans Health System and VA Eastern Colorado Health Care System. HJK was supported by NIH F99AG068438 and DBF by the Undergraduate Scholars Program, University of Florida. The work reported herein does not represent the views of the US Department of Veterans Affairs or the US government.

## Conflict of interest statement

The authors declare no conflict of interest.

## Supporting information


**Table S1.** Oligonucleotides used for RT‐qPCR.
**Table S2.** Antibodies used for immunofluorescence staining.
**Table S3.** Common DEG across all timepoints. Values are log2 fold change compared to SHAM. Red indicates upregulation and blue indicates downregulation. 1 W = 1 week, 2 W = 2 weeks, 1 M = 1 month, 3 M = 3 months.
**Table S4.** Top 5 Gene Ontology (biological process) of downregulated DEG.
**Figure S1.** Representative muscle fiber type images from 3 months SCI soleus stained with MHC I (blue), MHCIIa (red), MHCIIX (green), and Laminin (white), as described in the methods. Yellow fonts indicate different fiber types within the muscle.
**Figure S2.** Body weight (A) and locomotor function (B) data from SCI animals representing the 1 W, 2 W, and 1 M timepoints. Data presented are mean ± SEM. * *p* < 0.05 between SHAM and SCI, *p* < 0.05: ǂ vs. pre‐surgery, \ vs. 1 W, and θ vs. 2 W within group (Bonferroni's post‐hoc analysis). BBB: Basso‐Beattie‐Bresnahan.
**Figure S3.** Myofiber remodeling is accompanied by fiber type shift in SCI. (A) The median fiber area of Hybrid IIa/IIX, MHC IIX, Hybrid I/IIa/IIX, and Hybrid I/IIX from SCI soleus at 1 month (1 M) and 3 months (3 M) post‐injury. There were no MHC IIX positive fibers in SHAM muscles or in SCI at the 1 W and 2 W timepoints. (B) DEG that represent molecular mechanisms for the remodeling of cytoskeleton in the myofiber. Data presented for (A) are mean ± SEM. * *p* < 0.05 between 1 M and 3 M. Data presented for (B) are row z‐score where 0 represents the SHAM mean. DEG are indicated as SCI vs. SHAM: \ at 1 W, θ at 2 W, † at 1 M, and # at 3 M.
**Figure S4.** Representative images from the neuromuscular junction (NMJ) of SHAM and SCI soleus muscle 3 days postsurgery, stained with 2H3, ZNP‐1, SV2 (red), and α‐Bungarotoxin (green). Scale bars: 20 μm.

**Data S1.** Supporting Information.
