# Supplementary material for: Transcriptomics reveals transient and dynamic muscle fibrosis and atrophy differences following spinal cord injury in rats
Source: J Cachexia Sarcopenia Muscle. 2024 May 19;15(4):1309–23. doi: 10.1002/jcsm.13476 (PMC11294049; doi:10.1002/jcsm.13476)
Supplement: Supplementary file 1 — Table S1. Oligonucleotides used for RT‐qPCR. Table S2. Antibodies used for immunofluorescence staining. Table S3. Common DEG across all timepoints. Values are log2 fold change compared to SHAM. Red indicates upregulation and blue indicates downregulation. 1 W = 1 week, 2 W = 2 weeks, 1 M = 1 month, 3 M = 3 months. Table S4. Top 5 Gene Ontology (biological process) of downregulated DEG. Figure S1. Representative muscle fiber type images from 3 months SCI soleus stained with MHC I (blue), MHCIIa (red), MHCIIX (green), and Laminin (white), as described in the methods. Yellow fonts indicate different fiber types within the muscle. Figure S2. Body weight (A) and locomotor function (B) data from SCI animals representing the 1 W, 2 W, and 1 M timepoints. Data presented are mean ± SEM. * p < 0.05 between SHAM and SCI, p < 0.05: ǂ vs. pre‐surgery, \ vs. 1 W, and θ vs. 2 W within group (Bonferroni's post‐hoc analysis). BBB: Basso‐Beattie‐Bresnahan. Figure S3. Myofiber remodeling is accompanied by fiber type shift in SCI. (A) The median fiber area of Hybrid IIa/IIX, MHC IIX, Hybrid I/IIa/IIX, and Hybrid I/IIX from SCI soleus at 1 month (1 M) and 3 months (3 M) post‐injury. There were no MHC IIX positive fibers in SHAM muscles or in SCI at the 1 W and 2 W timepoints. (B) DEG that represent molecular mechanisms for the remodeling of cytoskeleton in the myofiber. Data presented for (A) are mean ± SEM. * p < 0.05 between 1 M and 3 M. Data presented for (B) are row z‐score where 0 represents the SHAM mean. DEG are indicated as SCI vs. SHAM: \ at 1 W, θ at 2 W, † at 1 M, and # at 3 M. Figure S4. Representative images from the neuromuscular junction (NMJ) of SHAM and SCI soleus muscle 3 days postsurgery, stained with 2H3, ZNP‐1, SV2 (red), and α‐Bungarotoxin (green). Scale bars: 20 μm. [file JCSM-15-1309-s001.pdf]

Supplemental Figure & Legends

Table S1. Oligonucleotides used for RT-qPCR.

**Gene**	**Primers (5'-3')**	
	**Sense**	**Antisense**
Rpl19	GTACCCTTCCTCTTCCCTATGC	CAATGCCAACTCTCGTCAACAG
Myh7	ACAGAGGAAGACAGGAAGAACCTAC	GGGCTTCACAGGCATCCTTAG
Myh2	TATCCTCAGGCTTCAAGATTTG	TAAATAGAATCACATGGGGACA
Myh1	CGCGAGGTTCACACCAAA	TCCCAAAGTCGTAAGTACAAAATGG
Myh4	CTGAGGAACAATCCAACGTC	TTGTGTGATTTCTTCTGTCACCT
Chrna1	GAGAAGATGACGCTGAGCATC	CGAACGGTGGTGTGTGTTGAT
Chrng	CAGAAGTGCACAGTGGCCACC	GGACACATTGAGCACGACCAC
Chrne	GAAATGCACGGTCTCTATCAACG	CGTTCGCAAAGATACGTTGAG

Table S2. Antibodies used for immunofluorescence staining.

**Antibody**	**Species**	**Source**	**Dilution**	**Product Number**
Primary				
Laminin	rabbit	Thermo Fisher	1:1000	RB-082-A
MHC I	mouse IgG2b	DSHB	1:50	BA-D5
MHC IIa	mouse IgG1	DSHB	1:50	SC-71
MHC IIX	mouse IgM	DSHB	1:20	6H1
Neurofilament	mouse IgG1	DSHB	1:150	2H3
Synaptotagmin 2	mouse IgG2a	DSHB	1:150	ZNP-1
Synaptic Vesicle Glycoprotein 2A	mouse IgG1	DSHB	1:100	SV2
Secondary (Invitrogen)				
Alexa Fluor 405 goat anti-rabbit			1:250	A-31556
Alexa Fluor 633 goat anti-mouse IgG2b			1:500	A-21146
Alexa Flour 594 goat anti-mouse IgG1			1:500	A-21125
Alexa Flour 488 goat anti-mouse IgM			1:500	A-21042
Alexa Flour 350 goat anti-mouse IgG2b			1:200	A-21140
Alexa Fluor 488 goat anti-rabbit			1:500	A-11070
Alexa Fluor 555 goat anti-mouse IgG			1:500	A-21422
Alexa Fluor 488 α-Bungarotoxin conjugate			1:500	B-13422

Table S3. Common DEG across all timepoints. Values are log2 fold change compared to SHAM. Red indicates upregulation and blue indicates downregulation. 1W = 1 week, 2W = 2 weeks, 1M = 1 month, 3M = 3 months.

Gene Symbol	1W	2W	1M	3M
Adcy2	1.36	2.00	1.29	1.43
Kcnq5	1.03	1.70	1.37	1.50
Mafa	1.55	2.73	2.32	1.77
Mylpf	2.05	3.60	3.13	5.20
Prkag3	1.42	2.80	2.05	1.78
Tmod1	1.80	2.14	1.47	1.42
Vwa3b	1.62	1.68	2.28	2.17
Bdh1	3.99	-2.99	-3.53	-4.91
Bbs9	-1.28	-1.22	-1.04	-1.34
Cacna1e	-5.48	-3.93	-4.67	-5.61
Cxcl12	-1.15	-1.58	-1.14	-1.06
Fabp3	-2.18	-1.82	-2.08	-1.24
Fzd9	-1.25	-1.15	-1.10	-1.24
Gnb3	-2.19	-1.74	-2.57	-2.65
Lrrc52	-4.20	-4.84	-3.38	-4.25
Oxct1	-1.40	-1.28	-1.14	-1.02
Slc4a3	-1.48	-1.25	-1.77	-3.56
Srrm3	-2.74	-2.11	-1.53	-2.03

Table S4. Top 5 Gene Ontology (biological process) of downregulated DEG.

ID	Description	p-value	p.adjust	q-value	Gene Count
1 Week					
GO:0033865	nucleoside bisphosphate metabolic process	8.598E-06	0.00539	0.00483	6
GO:0033875	ribonucleoside bisphosphate metabolic process	8.598E-06	0.00539	0.00483	6
GO:0034032	purine nucleoside bisphosphate metabolic process	8.598E-06	0.00539	0.00483	6
GO:0035383	thioester metabolic process	4.579E-05	0.01076	0.00965	5
GO:0045444	fat cell differentiation	0.0001568	0.03274	0.02936	7
2 Weeks					
GO:0015844	monoamine transport	4.637E-07	0.00149	0.00125	10
GO:0007599	hemostasis	1.981E-06	0.00151	0.00127	12
GO:0030198	extracellular matrix organization	2.599E-06	0.00151	0.00127	15
GO:0043062	extracellular structure organization	2.708E-06	0.00151	0.00127	15
GO:0045229	external encapsulating structure organization	2.822E-06	0.00151	0.00127	15
3 Months					
GO:0006937	regulation of muscle contraction	4.648E-09	2.1E-05	1.8E-05	24
GO:0006936	muscle contraction	1.077E-08	2.1E-05	1.8E-05	34
GO:0031589	cell-substrate adhesion	1.313E-08	2.1E-05	1.8E-05	37
GO:0030198	extracellular matrix organization	3.053E-08	2.5E-05	2.1E-05	32
GO:0043062	extracellular structure organization	3.306E-08	2.5E-05	2.1E-05	32

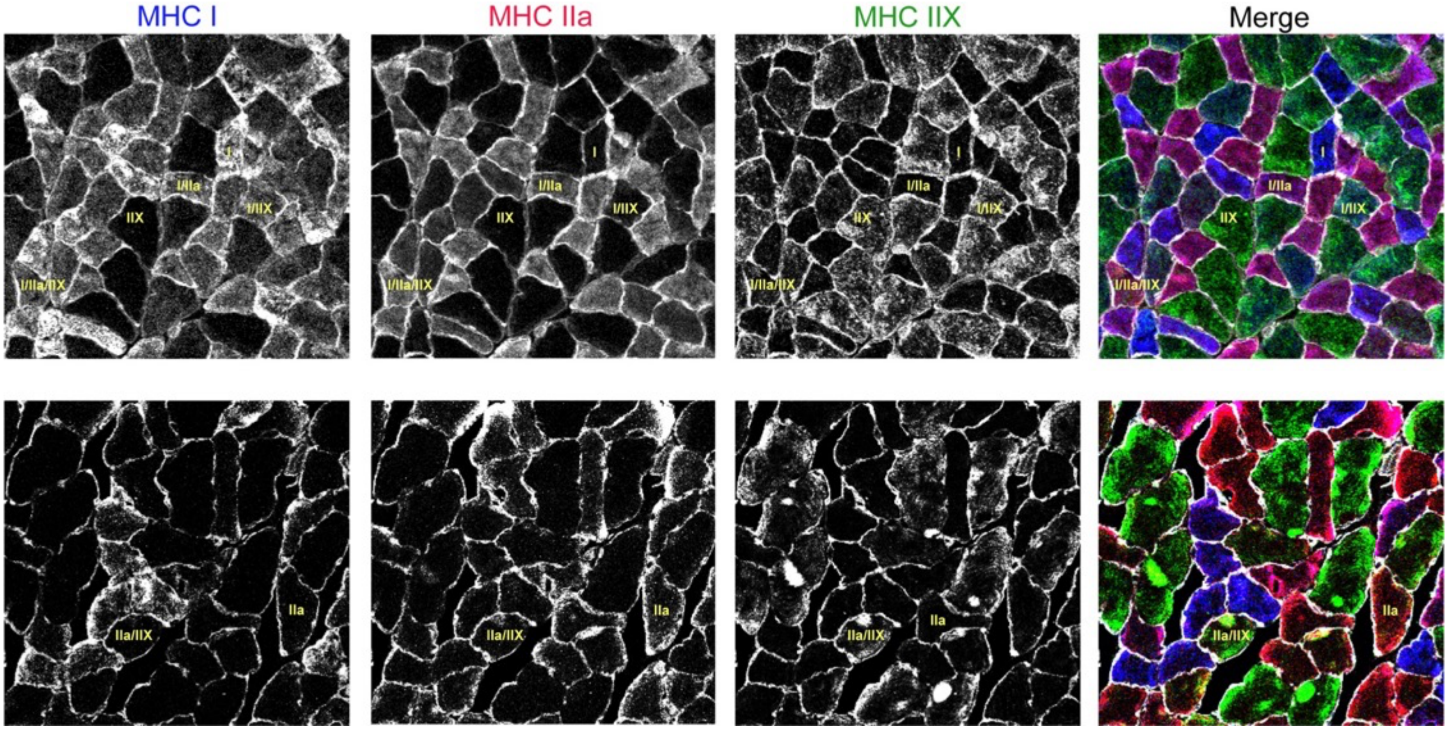

Figure S1

Representative muscle fiber type images from 3 months SCI soleus stained with MHC I (blue), MHCIIa (red), MHCIIX (green), and Laminin (white), as described in the methods. Yellow fonts indicate different fiber types within the muscle.

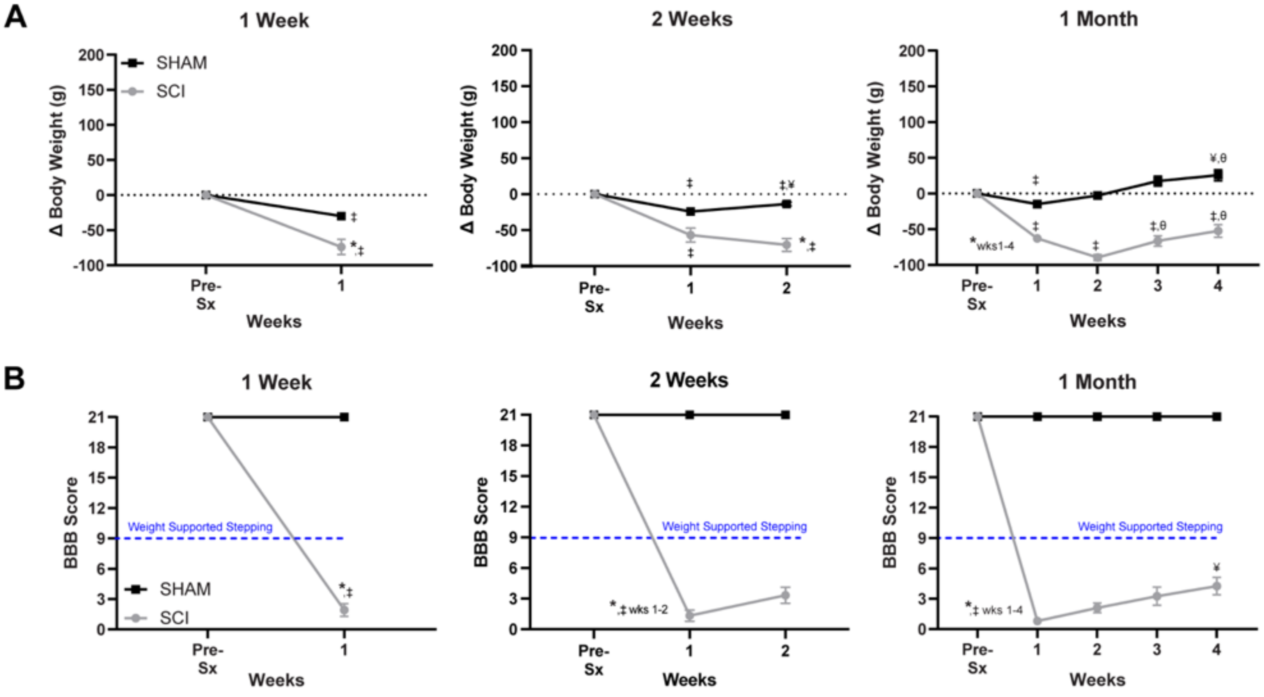

Figure S2

Body weight (A) and locomotor function (B) data from SCI animals representing the 1W, 2W, and 1M timepoints. Data presented are mean ± SEM. * $p < 0.05$ between SHAM and SCI, $p < 0.05$: ‡ vs. pre-surgery, ¥ vs. 1W, and θ vs. 2W within group (Bonferroni's post-hoc analysis). BBB: Basso-Beattie-Bresnahan.

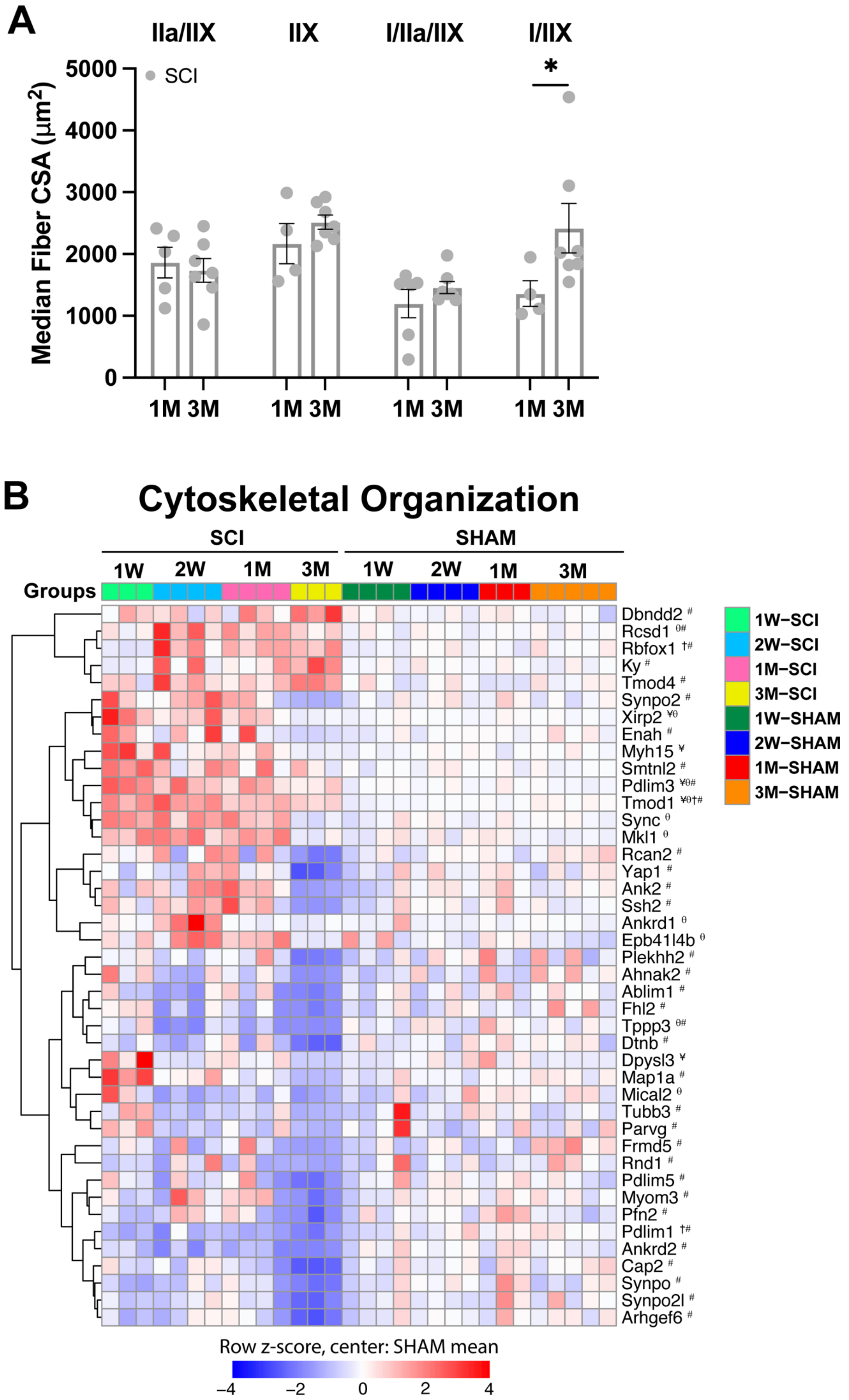

A
IIa/IIX
IIX
I/IIa/IIX
I/IIX
*
SCI
Median Fiber CSA (μm²)
5000
4000
3000
2000
1000
0
1M 3M
1M 3M
1M 3M
1M 3M
B
Cytoskeletal Organization
SCI
SHAM
1W
2W
1M
3M
1W
2W
1M
3M
Groups
Dbndd2 #
Rcsd1 θ#
Rbfox1 †#
Ky #
Tmod4 #
Synpo2 #
Xirp2 ¥θ
Enah #
Myh15 ¥
Smtnl2 #
Pdlim3 ¥θ#
Tmod1 ¥θ†#
Sync θ
Mkl1 θ
Rcan2 #
Yap1 #
Ank2 #
Ssh2 #
Ankrd1 θ
Epb41l4b θ
Plekhh2 #
Ahnak2 #
Ablim1 #
Fhl2 #
Tppp3 θ#
Dtnb #
Dpysl3 ¥
Map1a #
Mical2 θ
Tubb3 #
Parvg #
Frmd5 #
Rnd1 #
Pdlim5 #
Myom3 #
Pfn2 #
Pdlim1 †#
Ankrd2 #
Cap2 #
Synpo #
Synpo2l #
Arhgef6 #
1W–SCI
2W–SCI
1M–SCI
3M–SCI
1W–SHAM
2W–SHAM
1M–SHAM
3M–SHAM
Row z-score, center: SHAM mean
−4
−2
0
2
4

Figure S3

Myofiber remodeling is accompanied by fiber type shift in SCI. (A) The median fiber area of Hybrid IIa/IIX, MHC IIX, Hybrid I/IIa/IIX, and Hybrid I/IIX from SCI soleus at 1 month (1M) and 3 months (3M) post-injury. There were no MHC IIX positive fibers in SHAM muscles or in SCI at the 1W and 2W timepoints. (B) DEG that represent molecular mechanisms for the remodeling of cytoskeleton in the myofiber. Data presented for (A) are mean ± SEM. * $p < 0.05$ between 1M and 3M. Data presented for (B) are row z-score where 0 represents the SHAM mean. DEG are indicated as SCI vs. SHAM: ¥ at 1W, θ at 2W, † at 1M, and # at 3M.

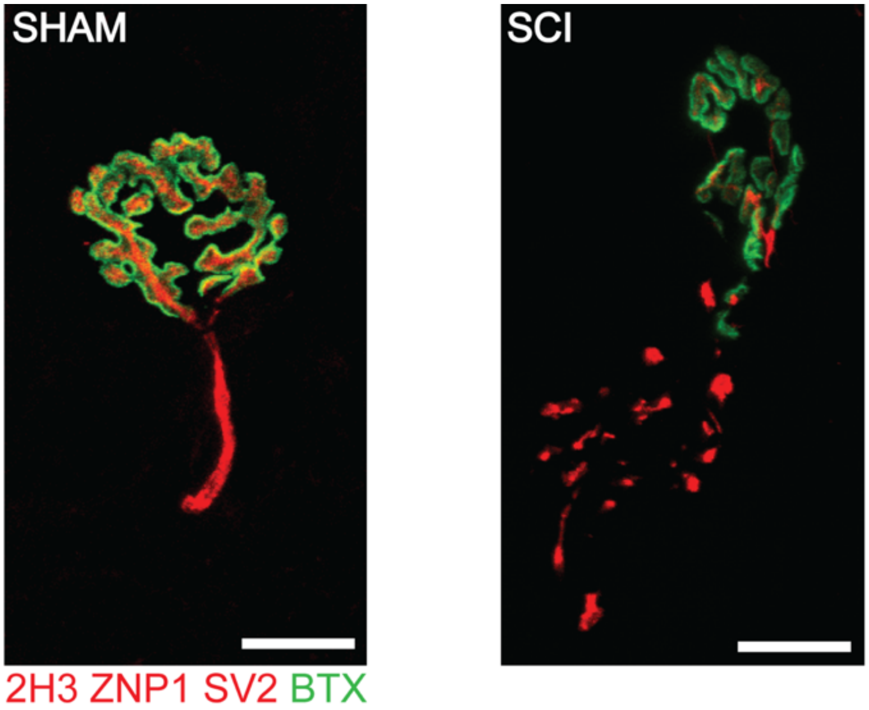

Figure S4

Representative images from the neuromuscular junction (NMJ) of SHAM and SCI soleus muscle 3 days post-surgery, stained with 2H3, ZNP-1, SV2 (red), and α-Bungarotoxin (green). Scale bars: 20 μm.
