# Supplementary material for: Transcriptomics reveals transient and dynamic muscle fibrosis and atrophy differences following spinal cord injury in rats
Source: J Cachexia Sarcopenia Muscle. 2024 May 19;15(4):1309–23. doi: 10.1002/jcsm.13476 (PMC11294049; doi:10.1002/jcsm.13476)
Supplement: Supplementary file 2 — Data S1. Supporting Information. [file JCSM-15-1309-s002.pdf]

SUPPLEMENTAL METHODS

Surgery, postoperative care, and tissue harvest

Barrier-free and specific-pathogen-free 4-month-old male Sprague-Dawley rats (n=49) were used. Animals were individually housed in a temperature-controlled animal facility with a 12 h light/dark cycle and *ad libitum* access to food and water. These rats were stratified according to their body weight into 1-week (1W), 2-weeks (2W), 1-month (1M), or 3-months (3M) groups and further randomized into the SHAM or SCI groups. All rats underwent a thoracic-level-9 (T_9) laminectomy to expose the spinal cord. In SCI animals, a severe contusion injury was produced by applying a 250-kilodyne force to the T_9 segment of the spinal cord via the Infinite Horizons Impactor (Precision Systems and Instrumentation). Subcutaneous injections of buprenorphine (0.05 mg/kg) and ketoprofen (5 mg/kg) were provided for 48 hours to all animals, along with 5 days of ampicillin treatment. Animals also received subcutaneous Ringer's saline solution to promote rehydration, along with Jell-O® mixture with added protein/fat, and apples to facilitate bodyweight maintenance. Manual bladder expressions were performed twice daily until spontaneous voiding is observed. Other postoperative care includes daily examinations for signs of distress, dehydration, weight loss, bladder dysfunction, fecal clearance, and skin lesions. Body weight was recorded every week, along with open-field locomotor function, which was assessed by two blinded observers using the Basso-Beattie-Bresnahan (BBB) locomotor rating scale [1]. At the end of the experiment, animals were euthanized, and the soleus muscles were excised and weighed. The left soleus muscles were snap frozen in liquid nitrogen, and the right soleus muscles were embedded in Tissue Tek® OCT (Sakura Finetek) and frozen in melting isopentane, then stored at -80°C.

SUPPLEMENTAL REFERENCES

1. Basso DM, Beattie MS, Bresnahan JC. Graded histological and locomotor outcomes after spinal cord contusion using the NYU weight-drop device versus transection. Experimental Neurology. 1996;139:244-56. doi:10.1006/exnr.1996.0098
